# Can AI singing retain users? Examining how experience, ethical acceptance, and affective engagement shape continuance intention

**DOI:** 10.3389/fpsyg.2026.1879311

**Published:** 2026-07-29

**Authors:** Yongxin Zhou, Yuhui Wang

**Affiliations:** 1 Department of Computer Science and Technology, Tsinghua University, Beijing, China; 2 Department of Voice and Opera, School of Music, Theatre & Dance, University of Michigan, Ann Arbor, MI, United States; 3 School of Architecture and Art, Central South University, Changsha, China

**Keywords:** affective engagement, AI singing, digital music participation, ethical acceptance, human-AI interaction

## Abstract

**Introduction:**

With the increasing integration of artificial intelligence into digital music applications, AI singing has emerged as a novel form of human–AI interaction in mobile karaoke platforms. Despite its growing popularity, limited research has examined the psychological mechanisms underlying users’ sustained engagement with AI singing, particularly from perspectives integrating affective experience and ethical perception. To address this gap, the present study aims to investigate the technological, affective, ethical, and agency-related factors influencing users’ continuance intention toward AI singing.

**Methods:**

Grounded in research on technology acceptance and psychological engagement, this study develops an extended framework incorporating Perceived Agency (PA), Ethical Acceptance (EA), and Affective Engagement (AE) to better explain users’ responses to AI-generated singing experiences. A total of 460 valid responses were collected through a survey, and structural equation modeling (SEM) was employed to test the proposed relationships.

**Results:**

The findings indicate that Performance Expectancy (PEF), Effort Expectancy (EE), Perceived Enjoyment (ENJ), Ethical Acceptance (EA), and Affective Engagement (AE) significantly and positively influence continuance intention. In contrast, Perceived Agency (PA), Perceived Innovativeness (PI), and Social Influence (SI) do not show significant effects.

**Discussion:**

The results suggest that sustained engagement with AI singing is shaped not only by functional evaluations, but also by emotional experience and ethical perception. This study contributes to research on psychological engagement in AI-enabled music interaction and provides practical implications for the design and optimization of AI singing applications.

## 1 Introduction

Artificial intelligence (AI) has increasingly become an important driving force in the development of digital music technologies. Early research on AI in music mainly focused on algorithmic composition and automatic music generation, employing various computational approaches such as neural networks, evolutionary algorithms and probabilistic models (Lopez-Rincon et al., 2018). More recent studies highlight that the integration of machine learning with music production has expanded beyond composition to include music information retrieval, recommendation systems and AI-assisted creative tools (Liang, 2023). These developments reflect the growing role of AI as both a technological infrastructure and a creative collaborator in digital music environments, among which AI singing has emerged as an important application that enables users to actively participate in AI-assisted musical performance.

Unlike AI music generation systems that primarily create musical content, AI singing focuses on synthesizing human singing voices based on musical scores and lyrics (Cho et al., 2021). Users are not only consumers of AI-generated outputs but also participants whose vocal identity may be represented, transformed, or recreated by the system. This process introduces psychological dimensions related to voice identity, self-expression, emotional performance, and perceived authenticity. Compared with traditional karaoke, where users directly perform songs themselves, AI singing enables users to augment or transform vocal performance through AI-generated voices, creating a unique form of human–AI co-performance (Reus, 2026).

Alongside advances in generative AI technologies, increasing scholarly attention has shifted toward understanding user experience and behavioral responses to AI-driven creative systems. Prior research suggests that human–AI collaborative outputs may receive higher aesthetic evaluations than purely human or AI-generated works, indicating the potential advantages of co-creative modes (Hitsuwari et al., 2023). Studies on AI-assisted music systems also report high levels of novelty and usability while identifying challenges related to user control and sustained engagement (Lee et al., 2025; Tchemeube et al., 2023). To explain such behaviors, established theoretical frameworks including the Expectation-Confirmation Model (ECM) and the Unified Theory of Acceptance and Use of Technology (UTAUT) have been widely applied in studies of AI adoption and continuance intention (Bhattacherjee, 2001; Chao, 2019; Pham et al., 2024; Wang et al., 2024). In highly interactive digital environments, users’ engagement with AI systems is often shaped not only by technological performance but also by emotional and ethical considerations. Prior studies indicate that anthropomorphic design and emotional expression can enhance user engagement and subjective experience (Ponce et al., 2025; Yu J. N. et al., 2024). In addition, interactive mechanisms such as gamification and real-time feedback may further stimulate affective involvement and participatory behavior (Boyle et al., 2016; Ge and Hu, 2025). At the same time, users increasingly form ethical judgments regarding AI systems, including concerns about transparency, fairness and trust, which may significantly influence technology acceptance and usage intention (Anton et al., 2021; Khan and Shehawy, 2025; Mustofa et al., 2025; Shao et al., 2025).

Despite the growing body of research on AI-enabled creative systems, empirical investigations into AI singing remain limited. AI singing integrates generative capabilities, affective expression and interactive platform environments, making it distinct from other AI applications. AI singing may be influenced not only by technological evaluations but also by emotional and identity-related perceptions. However, existing research has rarely examined how technological evaluations, emotional engagement and ethical perceptions jointly shape users’ continuance intention toward AI singing. Addressing this gap requires a multidimensional perspective that integrates technological experience, affective engagement and ethical acceptance.

Building upon the UTAUT, this study aims to investigate the technological, affective, ethical, and agency-related factors influencing users’ continuance intention toward AI singing. In addition to core constructs such as Performance Expectancy, Effort Expectancy, Social Influence and Perceived Enjoyment, the model introduces Affective Engagement, Ethical Acceptance, Perceived Agency and Perceived Innovativeness to better capture the characteristics of AI-driven creative interaction. Using survey data analyzed through covariance-based structural equation modeling (CB-SEM), this study investigates how technological, emotional and ethical factors jointly influence continuance intention toward AI singing.

Accordingly, this study addresses the following research questions:

RQ1: How do technological factors influence users’ continuance intention toward AI singing?
RQ2: How do affective engagement and ethical acceptance contribute to continuance intention toward AI singing?
RQ3: To what extent do perceived agency, perceived innovativeness, and social influence explain users’ continuance intention toward AI singing?

The remainder of this paper is organized as follows. Section 2 reviews relevant literature. Section 3 presents the research model and hypotheses. Section 4 describes the methodology. Section 5 reports the empirical results. Section 6 discusses the findings, and Section 7 concludes with theoretical and practical implications.

## 2 Literature review

### 2.1 User behavior research in music-related mobile applications

In recent years, with the rapid development of mobile music technologies, music-related applications have evolved from simple playback tools into multifunctional platforms integrating entertainment, social interaction and content recommendation. This transformation has attracted increasing scholarly attention to user behavior in digital music environments. Prior studies suggest that user engagement with music applications is shaped by multiple factors, including technology acceptance, platform design, personalized services and social interaction (Chandra et al., 2018; Yang and Teng, 2015).

Existing research on music streaming platforms has highlighted the importance of experiential and affective mechanisms in shaping user engagement. For example, personalization, satisfaction and usage habits have been found to significantly influence users’ continuance intention toward music services (Soaloon and Handayani, 2025). Other studies indicate that affective experiences, such as emotional resonance and social identification, may enhance users’ music exploration and participation in online music communities (Chen et al., 2024; Hesmondhalgh et al., 2024). Recent studies have also begun to explore user experiences in AI-supported music environments. For example, Wang and Liu (2025) found that positive experience significantly enhanced flow and learning efficiency in AI-based music education, highlighting the important role of affective and experiential processes in shaping users’ interactions with AI-assisted musical activities. These findings suggest that beyond functional utility, emotional and experiential factors may be central to understanding user engagement in emerging AI-driven music contexts. In addition, social video platforms have increasingly become important channels for music discovery, where algorithmic recommendation and social interaction influence patterns of music consumption and identity expression (Organ et al., 2025).

From a user experience perspective, previous research has also examined the design and functional attributes of music applications. Factors such as music diversity, interface usability and personalized services have been shown to significantly affect user satisfaction and overall platform experience (Zhou et al., 2022). Moreover, behavioral models such as the TAM and UTAUT have been widely applied to explain adoption and usage behavior in music-related technologies (Barata and Coelho, 2021; Zhou et al., 2025).

Despite the growing body of research on music streaming and discovery platforms, relatively limited attention has been paid to AI singing features that combine performative and creative elements. In particular, the mechanisms through which experiential, affective and ethical factors influence users’ continuance intention toward AI singing remain underexplored.

### 2.2 Theoretical foundations of continuance intention and extended variables

In information systems research, continuance intention has been widely regarded as a key indicator of users’ long-term usage behavior. Its theoretical foundation primarily derives from the ECM and its extensions. Bhattacherjee (2001) conceptualized continuance intention in information systems, proposing that users’ decisions to continue using a system after initial adoption are shaped by post-adoption evaluations. Subsequent studies have emphasized that confirmation, satisfaction and perceived usefulness are central determinants of continuance behavior (Halilovic and Cicic, 2013; Halilovic, 2015).

In mobile technology contexts, continuance intention has also been examined through integrated frameworks. For instance, Albashrawi (2021) combined ECM and TAM to investigate continuance intention in mobile banking systems, demonstrating that user satisfaction strongly predicts continued use, while perceived efficiency and ease of use serve as important antecedents. More recently, the Unified Theory of Acceptance and Use of Technology (UTAUT2) has shown strong explanatory power in continuance research. Studies applying UTAUT2 have found that factors such as habit, social influence and perceived value can significantly influence behavioral and continuance intentions in emerging technologies (Bahadur et al., 2024; Sergeeva et al., 2025).

In the context of music-related applications, continuance intention has been associated with both cognitive evaluations and affective experiences. For example, perceived usefulness, personalization and user satisfaction have been found to jointly influence continued use of music streaming platforms (Soaloon and Handayani, 2025). Beyond traditional acceptance variables, recent research has also begun to incorporate psychological and ethical factors into continuance models. Technology anxiety, privacy concerns and trust have been shown to shape users’ adoption and usage intentions in AI-driven systems (Ni and Cheung, 2023; Tao et al., 2025).

Overall, existing studies have primarily explained continuance intention through ECM and UTAUT-based frameworks, highlighting the roles of cognitive evaluation, affective experience and contextual factors in shaping users’ long-term technology use. These theoretical perspectives provide a useful foundation for examining continuance intention in emerging contexts such as AI singing.

## 3 Proposed model and hypotheses

This study examines users’ CI toward AI-powered singing within the broader context of generative AI applications in music platforms. To explain the multidimensional drivers of user behavior, an integrative research model is developed based on the UTAUT and its extension UTAUT2 (Venkatesh et al., 2003; Venkatesh et al., 2012). Core constructs including PEF, EE, SI, and ENJ are incorporated to capture both utilitarian and hedonic aspects of technology use.

**Figure 1:**
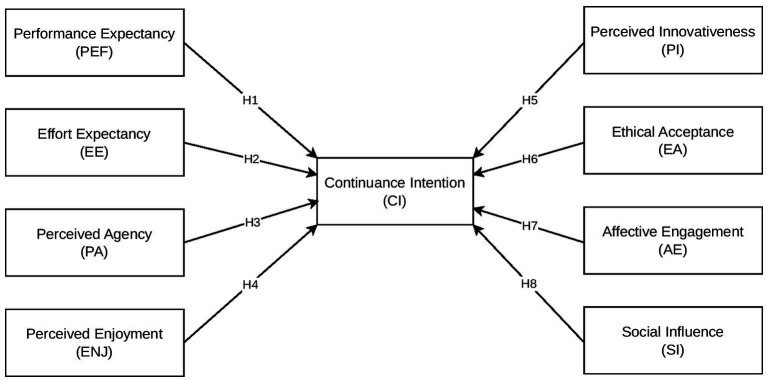
The proposed research model.

AI-powered singing integrates functional assistance, entertainment experience, and social interaction within karaoke environments. Users may evaluate whether the technology enhances vocal performance, improves recording quality, or simplifies operation, while also experiencing enjoyment and emotional immersion during interaction. In addition to the core UTAUT constructs, the model incorporates four additional variables, including PI, PA, EA, and AE. These variables capture the generative, interactive, and emotionally expressive characteristics of AI singing technologies. These variables extend the explanatory scope of the model by capturing users’ perceptions of novelty, autonomy, ethical legitimacy, and emotional involvement. The proposed conceptual model and hypotheses are presented in Figure 1.

### 3.1 Performance expectancy

Performance Expectancy (PEF) refers to the degree to which individuals believe that using a system enhances their performance (Venkatesh et al., 2003; Venkatesh et al., 2012). Prior studies consistently identify PEF as a key determinant of behavioral and continuance intentions across various technological contexts. Empirical research on generative AI systems indicates that expectations regarding system effectiveness and output quality significantly increase users’ adoption and continued usage intentions (Camilleri, 2024; Smutny and Sudzina, 2025).

In AI-powered singing applications, PEF reflects users’ beliefs that the technology can improve vocal accuracy, enhance expressive quality, or strengthen self-presentation in social karaoke environments. When users perceive that AI singing meaningfully improves their performance outcomes or interaction experiences, they are more likely to continue using the feature.

*H1*: Performance expectancy positively influences users’ continuance intention toward AI-powered singing.

### 3.2 Effort expectancy

Effort Expectancy (EE) refers to the degree of ease associated with using a system (Venkatesh et al., 2003; Venkatesh et al., 2012). Previous research has shown that perceived ease of use significantly influences both initial adoption and continuance intention across various digital technologies (Faradis and Afandi, 2025; Laradi et al., 2025).

In AI-powered singing applications, users may perceive the technology as technically complex due to processes such as voice modeling and algorithmic generation. When the interface is intuitive and the system is easy to operate, users experience lower cognitive effort and reduced technological anxiety. As a result, EE can facilitate sustained engagement with AI-powered singing functions.

*H2*: Effort expectancy positively influences users’ continuance intention toward AI-powered singing.

### 3.3 Perceived agency

Perceived Agency (PA) refers to the extent to which users attribute autonomy, intentionality, or decision-making capability to an AI system (Diwanji et al., 2025). In human-AI interaction research, higher levels of perceived agency may lead users to treat AI systems as quasi-autonomous collaborators rather than passive tools.

Previous studies suggest that perceptions of agency influence trust, emotional engagement, and cooperative behavior in human-AI collaboration contexts (Claure et al., 2025; Liao et al., 2025). In AI-powered singing environments, the technology simulates the creative act of a singer, which may encourage users to perceive the system as exhibiting a certain level of autonomy. When the AI is perceived as an active collaborator rather than merely a tool, users may also perceive its generated performances as creative and intentional. Such perceptions may enhance the interaction value of AI singing, thereby increasing users’ motivation to continue using the system (Liu et al., 2025). Therefore, perceived agency is expected to positively influence continuance intention toward AI-powered singing.

*H3*: Perceived agency positively influences users’ continuance intention toward AI-powered singing.

### 3.4 Perceived enjoyment

Perceived Enjoyment (ENJ) refers to the intrinsic pleasure derived from interacting with a technological system, independent of functional outcomes (Khedhaouria and Beldi, 2014). In technology acceptance research, ENJ has been widely identified as an important determinant of user motivation and continuance behavior, particularly in entertainment-oriented digital services.

Prior studies have consistently shown that ENJ positively influences continued use across various interactive technologies, including voice assistants, blogging platforms, and mobile learning systems (Shiau and Luo, 2013; Kowalczuk and Hof, 2025; Dou et al., 2025). In AI-powered singing applications, enjoyment may arise from the entertainment value of singing, the novelty of AI-generated vocal effects, and the expressive freedom offered by the platform. Such pleasurable experiences may strengthen users’ emotional attachment and motivate them to repeatedly engage with the feature.

*H4*: Perceived enjoyment positively influences users’ continuance intention toward AI-powered singing.

### 3.5 Perceived innovativeness

Perceived Innovativeness (PI) refers to users’ perception that a system embodies novelty, uniqueness, or innovative characteristics compared with existing alternatives (Watchravesringkan et al., 2010). In information systems and consumer research, PI has been associated with increased curiosity, perceived value, and adoption intention (Hong et al., 2017).

Empirical studies indicate that innovative technological features can enhance users’ perceptions of both utilitarian and hedonic value, thereby promoting continued use (Fauzi and Sheng, 2021; Ha et al., 2025). In generative AI environments, PI may also strengthen satisfaction and confirmation perceptions, which subsequently influence CI (Yu X. F. et al., 2024). When users perceive AI-powered singing as technologically novel and distinctive, their curiosity and experiential value may increase, encouraging continued engagement.

*H5*: Perceived innovativeness positively influences users’ continuance intention toward AI-powered singing.

### 3.6 Ethical acceptance

Ethical Acceptance (EA) refers to the extent to which users perceive a technology as morally appropriate and consistent with their ethical standards and social norms (Frhnel et al., 2024; Li et al., 2022). As AI increasingly participates in creative production, ethical considerations have become an important factor influencing users’ evaluations of AI systems.

Research on generative AI and deep synthesis technologies indicates that ethical perceptions affect technology acceptance through mechanisms such as perceived legitimacy and trust (Li et al., 2022). In AI-powered singing applications, ethical concerns may arise from issues such as voice data ownership, authorship attribution, and authenticity of artistic expression. When users perceive that the system operates transparently and respects creative originality, their trust and willingness to continue using the technology may increase.

*H6*: Ethical acceptance positively influences users’ continuance intention toward AI-powered singing.

### 3.7 Affective engagement

Affective Engagement (AE) reflects users’ emotional involvement during interaction with a system, including feelings of interest, immersion, and enjoyment (Hollebeek et al., 2014). As a key component of user engagement theory, AE has been linked to satisfaction, loyalty, and continued use of digital platforms.

Previous studies suggest that emotional engagement strengthens users’ psychological connection with digital systems and increases their willingness to interact with technology (Zhao and Ma, 2025; Urbonavicius et al., 2025). In AI-supported content generation contexts, positive emotional experiences such as curiosity and enjoyment may motivate long-term usage (Saeli and Rahmati, 2023). In AI-powered singing environments, immersive singing experiences and interactive feedback may enhance AE, thereby increasing CI.

*H7*: Affective engagement positively influences users’ continuance intention toward AI-powered singing.

### 3.8 Social influence

Social Influence (SI) refers to the degree to which individuals perceive that important others believe they should use a new system (Venkatesh et al., 2003). In technology adoption research, SI represents a key determinant of user behavior.

Previous studies indicate that peer pressure, group norms, and social identity can significantly influence technology adoption and continuance behaviors (Bozan et al., 2016; Xiao and Wang, 2016). In social platform environments such as karaoke applications, users’ behavior may be influenced by friends, online communities, and platform culture. Positive feedback and widespread adoption within social networks may therefore strengthen users’ motivation to continue using AI-powered singing.

*H8*: Social influence positively influences users’ continuance intention toward AI-powered singing.

## 4 Methodology

### 4.1 Research context

This study aims to investigate the technological, affective, ethical, and agency-related factors influencing users’ continuance intention toward AI singing. The research context is the AI singing function implemented in WeSing, one of the most widely used mobile karaoke applications in China. In 2025, the platform introduced an AI singing feature that allows users to generate personalized vocal models based on short recordings. By analyzing vocal timbre, pitch characteristics, and expressive features, the system can automatically generate singing performances in the user’s vocal style. This AI-generated singing environment provides an appropriate context for examining how technological experience, affective engagement, and ethical perception influence users’ continuance intention.

### 4.2 Measurement development

The questionnaire consisted of two parts. The first collected demographic information, including gender, age, education level, usage frequency, and level of music or singing training. The second measured the constructs included in the proposed model: performance expectancy (PEF), effort expectancy (EE), perceived agency (PA), perceived enjoyment (ENJ), perceived innovativeness (PI), ethical acceptance (EA), affective engagement (AE), social influence (SI), and continuance intention (CI).

Measurement items were adapted from validated scales in prior research to ensure content validity. PEF, EE, and SI were adapted from Venkatesh et al. (2003) and Venkatesh et al. (2012). PA was adapted from Taylor and Todd (1995) and Belanche et al. (2022). ENJ was adapted from Koufaris (2002) and Moon and Kim (2001). PI was adapted from Hwang et al. (2019). EA was adapted from Peca et al. (2016). AE was adapted from O'Brien and Toms (2010). CI was adapted from Bhattacherjee (2001), Bhattacherjee and Lin (2015), and Venkatesh et al. (2012). All items were measured using a seven-point Likert scale ranging from 1 (“strongly disagree”) to 7 (“strongly agree”). The final questionnaire included 33 items. The complete questionnaire is provided in Appendix A.

Prior to the main survey, the questionnaire was reviewed by five experts in entertainment software research to evaluate conceptual consistency and wording clarity. A pilot test with 50 respondents was subsequently conducted to assess item clarity and survey procedures. Minor wording adjustments were made based on the feedback obtained.

### 4.3 Data collection and sample

Participants were recruited through major Chinese social media platforms, including Rednote, Weibo, WeChat, and QQ. Before completing the survey, respondents were provided with a brief explanation of the study and were required to give informed electronic consent. Participants watched a short tutorial introducing the AI singing feature and were asked to experience the function before completing the questionnaire.

A total of 520 responses were initially collected. After excluding respondents who had not used AI singing and removing invalid responses (e.g., extremely short completion time, repetitive answers, or statistical outliers), 460 valid responses remained for analysis. The final sample size exceeded ten times the number of measurement items, satisfying the recommended threshold for reliable CB-SEM estimation (Hair et al., 2009; Hair et al., 2010).

The demographic characteristics of the participants are summarized in Table 1. The sample consisted of 242 males (52.6%) and 218 females (47.4%). Most respondents were aged between 25 and 44 years. The majority held a bachelor’s degree, and the largest proportion reported using AI singing one to two times per month. Regarding musical background, most participants indicated having a moderate level of prior music or singing experience.

**Table 1:** Descriptive statistics of the participants (*N* = 460).

Variable	Category	Frequency	Percent
Gender	Male	242	52.6
	Female	218	47.4
Age	18–24 years old	28	6.1
	25–34 years old	143	31.1
	35–44 years old	154	33.5
	45–54 years old	94	20.4
	Above 55 years old	41	8.9
Highest level of education	High school or below	82	17.8
	Associate degree	139	30.2
	Bachelor’s degree	173	37.6
	Master’s degree or above	66	14.4
Frequency of using the AI singing	Almost every day	16	3.5
	3–4 times per week	37	8.0
	1–2 times per week	94	20.4
	1–2 times per month	211	45.9
	Rarely use	102	22.2
Music/singing learning experience	None at all	10	2.2
	A little	125	27.2
	Moderate	141	30.6
	Quite a lot	116	25.2
	Very extensive	68	14.8

## 5 Results

This study employed SPSS v26 and AMOS v28 for statistical analysis. Data screening was completed prior to analysis, and all invalid responses had been removed. The normality of the data was assessed using the Jarque-Bera test. Following the guideline of Tabachnick et al. (2007), the absolute values of skewness and kurtosis were below 2.58 for all measurement items, indicating that the data satisfied the assumption of normality and were appropriate for subsequent confirmatory factor analysis (CFA) and structural equation modeling (SEM). Descriptive statistics are presented in Table 2.

**Table 2:** Mean value, standard deviations, and normality test of the items.

Constructs	Item	M	SD	Skewness	Kurtosis
Performance Expectancy (PEF)	PEF1	4.709	1.593	−0.379	−0.715
	PEF2	4.707	1.589	−0.217	−0.941
	PEF3	4.722	1.606	−0.332	−0.811
	PEF4	4.680	1.601	−0.400	−0.653
	PEF5	4.707	1.645	−0.308	−0.855
Effort Expectancy(EE)	EE1	4.678	1.664	−0.290	−0.958
	EE2	4.654	1.592	−0.387	−0.762
	EE3	4.717	1.623	−0.485	−0.650
	EE4	4.765	1.560	−0.460	−0.545
Perceived Agency (PA)	PA1	4.576	1.614	−0.247	−1.028
	PA2	4.683	1.588	−0.299	−0.760
	PA3	4.646	1.636	−0.353	−0.745
	PA4	4.672	1.606	−0.337	−0.849
Perceived Enjoyment (ENJ)	ENJ1	4.850	1.523	−0.407	−0.604
	ENJ2	4.650	1.539	−0.301	−0.705
	ENJ3	4.741	1.555	−0.359	−0.717
	ENJ4	4.846	1.547	−0.432	−0.647
Perceived Innovativeness (PI)	PI1	4.574	1.665	−0.255	−1.014
	PI2	4.604	1.556	−0.249	−0.844
	PI3	4.598	1.627	−0.313	−0.816
Ethical Acceptance (EA)	EA1	4.789	1.600	−0.406	−0.734
	EA2	4.763	1.636	−0.412	−0.719
	EA3	4.865	1.592	−0.568	−0.493
	EA4	4.815	1.548	−0.443	−0.614
Affective Engagement (AE)	AE1	4.746	1.563	−0.332	−0.794
	AE2	4.752	1.610	−0.410	−0.748
	AE3	4.733	1.534	−0.275	−0.779
Social Influence (SI)	SI1	4.778	1.576	−0.352	−0.762
	SI2	4.774	1.554	−0.417	−0.694
	SI3	4.696	1.612	−0.381	−0.851
Continuance Intention (CI)	CI1	4.854	1.535	−0.487	−0.574
	CI2	4.880	1.531	−0.492	−0.552
	CI3	4.852	1.588	−0.467	−0.708

### 5.1 Measurement model assessment

The reliability and validity of the measurement model were evaluated through CFA. According to established criteria, factor loadings should exceed 0.70, composite reliability (CR) and Cronbach’s *α* should exceed 0.70, and the average variance extracted (AVE) should exceed 0.50 (Fornell and Larcker, 1981; Hair et al., 2010). As shown in Table 3, Cronbach’s α values ranged from 0.859 to 0.893, indicating strong internal consistency. All CR values exceeded 0.70 and AVE values exceeded 0.50, demonstrating satisfactory convergent validity. In addition, the standardized factor loadings of all items were above 0.70. Discriminant validity was assessed using the Fornell-Larcker criterion. As reported in Table 4, the square root of the AVE for each construct exceeded its correlations with other constructs, confirming adequate discriminant validity.

**Table 3:** Structural reliability and convergent validity.

Constructs	Items	Cronbach’s alpha	Standardized factor loading	CR	AVE
Performance Expectancy (PEF)	PEF1	0.900	0.801	0.900	0.643
	PEF2		0.808		
	PEF3		0.803		
	PEF4		0.814		
	PEF5		0.784		
Effort Expectancy (EE)	EE1	0.883	0.802	0.883	0.654
	EE2		0.795		
	EE3		0.813		
	EE4		0.824		
Perceived Agency (PA)	PA1	0.879	0.810	0.879	0.644
	PA2		0.813		
	PA3		0.785		
	PA4		0.802		
Perceived Enjoyment (ENJ)	ENJ1	0.856	0.758	0.857	0.599
	ENJ2		0.766		
	ENJ3		0.777		
	ENJ4		0.796		
Perceived Innovativeness (PI)	PI1	0.850	0.803	0.851	0.656
	PI2		0.834		
	PI3		0.794		
Ethical Acceptance (EA)	EA1	0.867	0.787	0.867	0.620
	EA2		0.785		
	EA3		0.796		
	EA4		0.782		
Affective Engagement (AE)	AE1	0.831	0.786	0.831	0.621
	AE2		0.796		
	AE3		0.782		
Social Influence (SI)	SI1	0.832	0.810	0.833	0.624
	SI2		0.776		
	SI3		0.784		
Continuance Intention (CI)	CI1	0.835	0.773	0.835	0.628
	CI2		0.804		
	CI3		0.800		

**Table 4:** Discriminant validity: Pearson correlation and AVE square root values.

Constructs	PEF	EE	PA	ENJ	PI	EA	AE	SI	CI
PEF	**0.802**								
EE	0.431	**0.809**							
PA	0.463	0.455	**0.802**						
ENJ	0.439	0.460	0.422	**0.774**					
PI	0.422	0.412	0.454	0.397	**0.810**				
EA	0.488	0.457	0.478	0.371	0.430	**0.787**			
AE	0.455	0.465	0.417	0.366	0.398	0.450	**0.788**		
SI	0.480	0.465	0.393	0.411	0.351	0.467	0.407	**0.790**	
CI	0.511	0.495	0.474	0.487	0.421	0.486	0.481	0.451	**0.792**

The bold items on the diagonal represent the square roots of the AVE, off-diagonal elements are the correlation estimates.

Since all variables were measured using self-reported questionnaires collected at a single time point, Harman’s single-factor test was conducted to assess potential common method bias. All measurement items were entered into an exploratory factor analysis without rotation. The first unrotated factor accounted for 37.29% of the total variance, which is below the commonly accepted threshold of 40% (Podsakoff et al., 2003), indicating that common method bias was unlikely to be a serious concern in this study.

Model fit was evaluated using multiple goodness-of-fit indices. The results indicate that the model achieved a satisfactory fit (χ^2^/df = 1.178, RMSEA = 0.020, SRMR = 0.029, CFI = 0.990, TLI = 0.989), all meeting the recommended thresholds (Hair et al., 2010). Detailed results are presented in Table 5. It should be noted that no correlated error terms or post-hoc model modifications were introduced during CFA or SEM estimation. The reported fit indices were obtained from the originally specified theoretical model.

**Table 5:** Model fit indices.

Fit index	Recommended value (Hair et al., 2006; Hu and Bentler, 1999)	Obtained
χ2	Non-significant at *p* < 0.05	540.750
df	n/a	459
χ2/df	preferable <3	1.178
GFI	>0.90	0.935
AGFI	>0.80	0.921
CFI	>0.90	0.990
RMSEA	<0.08	0.020
SRMR	<0.08	0.029
TLI	>0.90	0.989
NFI	>0.90	0.940
NNFI	>0.90	0.989

### 5.2 Path analysis and hypothesis validation

**Figure 2:**
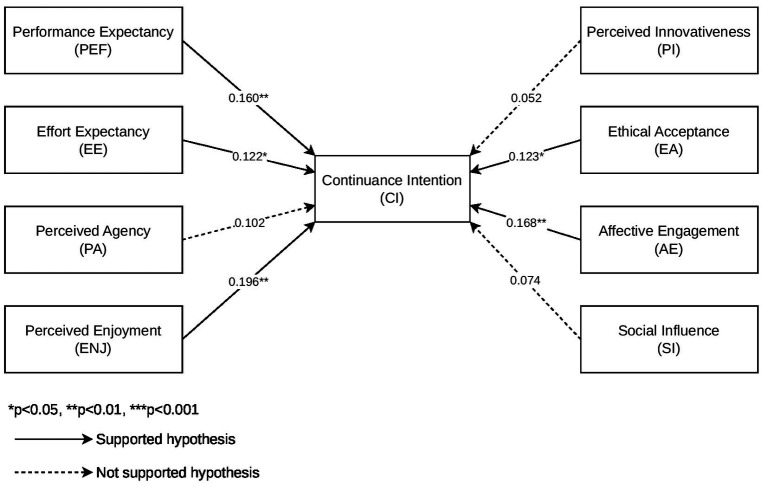
Research model with standardized path coefficients.

The structural model was tested using SEM. The results show that five hypotheses were supported while three were not. As presented in Table 6 and Figure 2, PEF, EE, ENJ, EA, and AE significantly influenced CI, supporting H1, H2, H4, H6, and H7.

**Table 6:** Hypothesis testing.

Hypothesis	Standardized coefficient	S.E.	C.R.	Status
H1: PEF → CI	0.160**	0.055	2.690	Supported
H2: EE → CI	0.122*	0.054	2.025	Supported
H3: PA → CI	0.102	0.053	1.743	Not supported
H4: ENJ → CI	0.196**	0.059	3.427	Supported
H5: PI → CI	0.052	0.050	0.922	Not supported
H6: EA → CI	0.123*	0.058	1.993	Supported
H7: AE → CI	0.168**	0.058	2.782	Supported
H8: SI → CI	0.074	0.057	1.215	Not supported

⁎*p* < 0.05; ⁎⁎*p* < 0.01; ⁎⁎⁎*p* < 0.001.

Among these predictors, ENJ had the strongest effect on CI (*β* = 0.196, *p* < 0.01), followed by AE (*β* = 0.168, *p* < 0.01) and PEF (*β* = 0.160, *p* < 0.01). In contrast, PA (*β* = 0.102, *p* > 0.05), PI (*β* = 0.052, *p* > 0.05), and SI (*β* = 0.074, *p* > 0.05) did not significantly predict CI, leading to the rejection of H3, H5, and H8. These findings suggest that users’ continuance intention toward AI singing is primarily driven by experiential, emotional, and ethical factors rather than perceived innovativeness, agency, or external social influence.

### 5.3 Demographic moderation analyses

To examine whether the determinants of continuance intention differ across user groups, moderation analyses were conducted based on gender, age, education level, usage frequency, and music training background.

The results of the demographic moderation analyses are summarized in Table 7. Overall, gender and music training background did not significantly moderate the relationships in the model. Likewise, age showed no significant moderating effects, although the interaction between AE and age approached significance (*β* = 0.097, *p* = 0.069), suggesting that AE may play a relatively stronger role among users aged 35 years and above.

**Table 7:** Summary of demographic moderation analyses.

Moderator	Grouping criteria	Significant relationship	β	*p*	Interpretation
Gender	Male vs. Female	None	−	>0.05	No significant moderation
Age	18–34 vs. ≥ 35 years	AE × Age → CI	0,097	0.069	Marginal
Education	Below bachelor’s degree vs. Bachelor’s degree or above	EE × Education → CI	−0.124	0.037*	Significant
Usage frequency	High (≥ 3 times/week) vs. Low (≤2 times/week)	EE × frequency → CI	0.352	0.005*	Significant
		AE × frequency → CI	0.209	0.056	Marginal
Music training	Basic or below vs. above basic	None	−	>0.05	No significant moderation

Regarding education level, a significant interaction effect was observed between EE and education (*β* = −0.124, *p* = 0.037), indicating that the positive influence of EE on CI was stronger among users with a bachelor’s degree or below than among those with higher educational attainment. This finding suggests that ease of use remains particularly important for users with lower educational attainment.

Usage frequency also moderated the relationship between EE and CI. Specifically, the interaction between EE and usage frequency was significant (*β* = 0.352, *p* = 0.005), indicating that effort expectancy exerted a stronger influence on continuance intention among low-frequency users than among high-frequency users. In addition, the interaction between AE and usage frequency approached significance (*β* = 0.209, *p* = 0.056), suggesting that emotional engagement may also facilitate the transition from occasional use to sustained engagement.

## 6 Discussion

This study predicted CI toward the AI singing function based on an extended UTAUT framework. Through survey data collected from WeSing users, the results show that five out of the eight proposed hypotheses were supported. Specifically, PEF and EE from the UTAUT model, together with ENJ, EA, and AE, exerted significant positive effects on CI, while PA, PI, and SI did not significantly predict CI. In addition, the moderating effects of gender, age, education level, usage frequency, and music training experience were examined.

### 6.1 Performance expectancy, effort expectancy, and perceived agency

The findings indicate that both PEF and EE have significant positive effects on users’ continuance intention, confirming the explanatory power of the UTAUT framework in entertainment-oriented and creative digital contexts. Even in karaoke applications primarily driven by entertainment and emotional experience, users’ decisions to continue using a technological function still depend strongly on whether the function enhances performance and whether it is easy to use.

The significant influence of PEF suggests that users do not regard AI singing merely as a novel entertainment feature, but associate it with improvements in their singing performance. When users perceive that AI singing can assist with pitch accuracy, rhythm alignment, emotional expression, or overall performance quality, their motivation may shift from short-term trial to long-term reliance. Prior research in multiple domains, including accounting information systems, museum guidance systems, and wearable health technologies, has consistently shown that performance expectancy effectively predicts continuance behavior (Liang and Wang, 2026; Lutfi, 2022; Tian et al., 2025). In the context of AI singing, when users perceive that the technology meaningfully improves vocal output and content quality, their willingness to continue using the function naturally increases.

EE also shows a significant positive effect on CI, indicating that acceptance of AI singing depends not only on technological sophistication but also on whether the operation process is cognitively manageable. Although AI singing involves complex mechanisms such as algorithmic generation and audio processing, users’ continuance intention increases when interaction processes are simplified and learning barriers are reduced. In entertainment applications, ease of use is closely tied to overall user experience, and excessive operational complexity may discourage continued use even when the technology itself is valuable (Liang and Wang, 2026). Similar findings have been reported in studies of intelligent systems (Lutfi, 2022), virtual learning platforms (Li et al., 2025), and mobile banking services (Dang et al., 2024).

Interestingly, PA did not significantly predict CI. One possible explanation is that PA may not directly motivate sustained use but instead operates through more proximal psychological mechanisms. Previous studies have suggested that attributing autonomy or intentionality to AI systems primarily enhances users’ trust, emotional engagement, and collaborative experience rather than directly influencing behavioral intention (Claure et al., 2025; Liao et al., 2025). In AI singing, perceiving the system as an autonomous creative collaborator may first shape users’ psychological experiences during interaction rather than directly determining continuance intention. When users perceive AI-generated performances as intentional and creative rather than purely algorithmic outputs, the interaction may become more enjoyable because the AI is experienced as an active co-performer rather than a passive tool. This perception may also foster stronger affective engagement by creating a greater sense of emotional involvement and collaborative participation during the singing experience. Furthermore, attributing autonomy and competence to the AI may strengthen users’ trust in the quality and reliability of AI-generated performances, increasing confidence in the system’s creative capability. These positive psychological responses may subsequently translate into stronger continuance intention. Therefore, the non-significant direct effect of PA observed in the present study may reflect an indirect mechanism that warrants further investigation. Therefore, the non-significant direct effect observed in the present study may reflect an indirect mechanism that warrants further investigation.

From the perspective of entertainment platforms, these results extend the applicability of UTAUT beyond purely utilitarian contexts. In AI-assisted singing, which combines instrumental functionality with expressive creativity, users’ evaluations of usefulness and operational convenience remain fundamental drivers of continuance intention. This finding aligns with evidence from AI music generation research indicating that technological usefulness and ease of interaction continue to shape user engagement even in creative AI applications (Zhou et al., 2025).

### 6.2 Perceived enjoyment, ethical acceptance, and affective engagement

The results further indicate that ENJ, EA, and AE significantly influence users’ continuance intention. These findings suggest that continued use of AI singing is shaped not only by technological usefulness but also by emotional experience, value alignment, and affective involvement.

First, perceived enjoyment plays an important role in sustaining engagement with AI singing. Enjoyment reflects the positive emotional experience generated during interaction and has frequently been identified as a key factor influencing behavioral intention in hedonic systems. Studies in music education and interactive systems suggest that enjoyment can directly influence behavioral intention and may also mediate relationships between interactive experience and technology adoption (Li, 2025). In continuance research, enjoyment is often conceptualized as a form of positive affect that contributes to satisfaction, which is a major predictor of continuance intention (Pereira and Tam, 2021). Within karaoke applications, when users experience pleasurable outcomes such as improved vocal output, enhanced production quality, or satisfaction when sharing performances, continued use may gradually become driven by repeated emotional reward rather than purely functional considerations. Similar effects of enjoyment have been identified in mobile AR entertainment (Hung et al., 2021) and music streaming services (Talledo et al., 2023). Beyond enjoyment itself, the present findings can also be interpreted from the perspective of flow theory. Previous research suggests that positive experiences facilitate flow experiences, which in turn enhance learning outcomes in AI-supported music environments (Wang and Liu, 2025). AI singing similarly provides users with immediate feedback, creative expression, and continuous interaction, all of which are conducive to immersive experiences. Such flow experiences may strengthen users’ affective engagement with AI singing and reinforce their motivation to continue using the technology. This perspective further suggests that the significant effects of ENJ and AE observed in the present study may partly reflect the psychological benefits associated with immersive human–AI interaction.

Second, ethical acceptance significantly predicts continuance intention. In AI singing contexts where technology effectively performs on behalf of the user, individuals may evaluate whether the system is legitimate, trustworthy, and socially acceptable. Ethical considerations related to voice synthesis, identity representation, and creative ownership therefore become important psychological factors influencing continued use. Prior research shows that societal acceptance of AI systems is shaped by ethical perception, transparency, and responsibility attribution (Lin and Chung, 2025). When users perceive AI systems as transparent and responsible in their use of data and generation processes, acceptance increases, which in turn strengthens continuance intention. Ethical perception may also influence continued use indirectly through trust formation (Yang et al., 2025).

Finally, the significant effect of AE highlights the role of emotional investment in AI entertainment applications. Continuance intention often depends on whether users develop psychological attachment and emotional connection through repeated interaction. Affective engagement can enhance immersion, belonging, and self-expression, thereby strengthening users’ long-term relationship with digital systems (Shang et al., 2026). In AI singing, users repeatedly listen to, compare, and internalize AI-generated vocal performances, forming emotional connections with the synthesized voice. Research in human–computer interaction and organizational psychology suggests that affective investment can strongly predict long-term behavioral intention (Fukuzaki et al., 2023; Trudeau et al., 2019).

Overall, the effects of ENJ, EA, and AE suggest that continuance of AI singing involves a complex psychological process integrating emotional experience, ethical evaluation, and value alignment. By incorporating these dimensions, the present study extends the traditional UTAUT framework and reflects the broader shift in AI acceptance research from purely functional explanations toward more integrated emotional and ethical perspectives (Lin and Chung, 2025; Salam et al., 2026).

### 6.3 Demographics

Moderation analyses were conducted to examine the moderating roles of demographic characteristics, including gender, age, education level, usage frequency, and music training background. Overall, the results indicated that demographic characteristics exerted only limited moderating effects, suggesting that the continuance mechanism of AI singing remains relatively stable across different user groups. This finding is broadly consistent with the theoretical assumptions of UTAUT2, which propose that demographic characteristics primarily function as boundary conditions rather than direct determinants of behavioral intention (Venkatesh et al., 2012).

Specifically, neither gender nor music training background significantly moderated the proposed relationships. Likewise, age showed only a marginal moderating effect on the relationship between AE and CI. This result reflects the broader trend that the influence of traditional demographic characteristics on technology acceptance has gradually weakened as intelligent technologies become increasingly accessible. In entertainment-oriented digital environments, user experiences tend to become more homogeneous across demographic groups. Nevertheless, the marginal interaction between age and AE suggests that emotional engagement may become relatively more important for older users, who may place greater emphasis on emotional expression and psychological fulfillment when interacting with AI-supported entertainment technologies.

Education level significantly moderated the relationship between EE and CI. Specifically, the positive influence of EE became weaker as educational attainment increased, indicating that ease of use was particularly important for users with lower educational attainment. This finding suggests that users with lower educational backgrounds may be more sensitive to the learning costs associated with emerging AI technologies, whereas users with higher educational attainment may place relatively greater emphasis on experiential, functional, or value-related benefits once basic usability requirements have been satisfied.

Usage frequency also moderated the influence of EE on CI. The stronger effect of EE among low-frequency users suggests that ease of use plays a particularly important role during the early stages of adoption, when users are still evaluating whether AI singing is worth continued use. In contrast, the absence of significant moderation among high-frequency users may reflect the formation of habitual usage patterns, reducing the relative importance of usability. Furthermore, the marginal moderating effect of AE implies that positive emotional experiences may facilitate the transition from occasional use to sustained engagement.

Overall, the demographic analyses suggest that CI toward AI singing is primarily driven by experiential, emotional, and value-based perceptions rather than demographic characteristics. Although education level and usage frequency moderated specific relationships, these effects did not substantially alter the overall structural relationships proposed in the model, supporting the robustness and generalizability of the proposed framework.

## 7 Conclusion, limitations, and future work

This study investigated the technological, affective, ethical, and agency-related factors influencing users’ continuance intention toward AI singing and developed a multidimensional model based on an extended UTAUT framework. Using 460 valid responses analyzed through CB-SEM, the results indicate that PEF, EE, ENJ, EA, and AE significantly and positively predict CI. Among these factors, ENJ exerts the strongest effect, followed by AE and PEF, while PA, PI, and SI do not reach statistical significance. Moderation analyses further shows that most demographic characteristics, including gender, age, and music training background, do not significantly moderate the core relationships, suggesting that the continuance mechanism of AI singing remains relatively stable across user groups. However, users with lower education levels and lower usage frequency appear more sensitive to ease of use and affective factors, indicating that lowering operational barriers and strengthening emotional engagement may help promote sustained usage.

From a theoretical perspective, this study extends continuance intention research by introducing AI singing as a representative context of generative entertainment technology. The results confirm the applicability of key UTAUT constructs such as PEF and EE in AI entertainment applications, while demonstrating that ethical acceptance and affective engagement provide additional explanatory power beyond traditional technology acceptance variables. The findings suggest that continuance intention toward AI singing emerges from the combined influence of functional evaluation, emotional experience, and value-based judgment, thereby offering a more comprehensive framework for understanding the long-term adoption of generative music technologies.

Methodologically, this study employs CB-SEM to test multivariate path relationships and integrates moderation analyses across demographic and usage-related variables. By combining structural equation modeling with demographic moderation analyses, the study provides a systematic approach for examining continuance mechanisms and potential boundary conditions in AI-based entertainment technologies.

From a practical perspective, the results indicate that promoting users’ continuance intention toward AI singing features requires more than technological optimization alone. In addition to enhancing system performance and usability, developers should also consider users’ emotional experiences and ethical perceptions during interaction. Mechanisms such as copyright disclosure, data-use transparency, explainability of AI-generated results, and clear labeling of AI-generated content may help transform novelty-driven trial into stable and sustained usage.

Despite these contributions, several limitations should be acknowledged. First, the study adopts a cross-sectional survey design, which limits the ability to capture the dynamic evolution of user attitudes and behaviors over time. Future research could employ longitudinal or experimental designs to examine how affective engagement, ethical perceptions, and continuance intention evolve across different stages of technology use.

Second, the study focuses on a single platform and primarily samples Chinese mobile karaoke users. Although this context provides strong ecological validity, the generalizability of the findings to other cultural settings or other AI music applications, such as AI composition or AI accompaniment, requires further investigation. In addition, participants were recruited through major social media platforms, which may have introduced self-selection bias. Individuals who are more interested in AI technologies, digital music, or karaoke applications may have been more likely to participate in the survey. Therefore, caution should be exercised when generalizing the findings to broader populations of AI music users.

Third, although ethical acceptance and affective engagement were incorporated to extend the explanatory scope of UTAUT, additional psychological and cultural variables may also influence long-term usage decisions. Factors such as artistic identity, perceptions of originality and creative ownership, and concerns about algorithmic bias may further shape users’ attitudes toward generative AI technologies.

Finally, all variables were measured using self-reported questionnaires collected at a single time point. Although common method bias was assessed and did not appear to pose a serious concern, self-reported measures may still be influenced by social desirability and subjective evaluation biases. Furthermore, objective platform usage records were not available. Future studies may combine survey measures with behavioral log data to provide a more comprehensive understanding of actual usage patterns and continuance behavior.

Future research may extend this study in several directions. Longitudinal designs or platform behavioral data could be used to examine how experiential factors such as enjoyment and affective engagement translate into actual continuance behavior over time. Researchers may also explore potential mediation mechanisms among technological cognition, emotional experience, and value-based judgments. For example, PEF and EE may influence CI indirectly through enjoyment or affective engagement, while ethical acceptance may operate through mediators such as trust, perceived risk, or AI anxiety. In addition, ethical acceptance could be further decomposed into more specific dimensions, including copyright attribution, privacy and data usage, algorithmic transparency, fairness, and potential misuse risks. Comparing the relative influence of these ethical factors may provide deeper insight into how users evaluate generative AI entertainment technologies.

Finally, future studies could expand the research context to multiple platforms and technological implementations. AI singing systems may differ in design features such as voice cloning, pitch correction, style transfer, or virtual avatar singing. Comparing these different implementations, as well as conducting cross-cultural analyses, would help clarify how emotional and ethical perceptions shape continuance intention in diverse technological and cultural environments.

## Data Availability

The raw data supporting the conclusions of this article will be made available by the authors, without undue reservation.
