# Supplementary material for: Can AI singing retain users? Examining how experience, ethical acceptance, and affective engagement shape continuance intention
Source: Front Psychol. 2026 Jul 29;17:1879311. doi: 10.3389/fpsyg.2026.1879311 (PMC13463292; doi:10.3389/fpsyg.2026.1879311)
Supplement: Supplementary file 1 [file Supplementary_file_1.pdf]

Appendix A

Structures	Items	Statements	References
Performance Expectancy (PEF)	PEF1	I believe that using AI singing enables me to perform singing-related activities more conveniently.	Venkatesh et al. (2003, 2012)
	PEF2	I believe that using AI singing allows me to sing at different times and locations.	
	PEF3	I believe that using AI singing makes it easier for me to complete singing tasks.	
	PEF4	I believe that using AI singing improves my efficiency in recording songs.	
	PEF5	I believe that using AI singing enhances my overall singing experience.	
Effort Expectancy (EE)	EE1	I believe that the interface of AI singing is clear and understandable.	Venkatesh et al. (2003, 2012)
	EE2	I believe that AI singing is easy to use.	
	EE3	I believe that most functions of AI singing are easy to learn.	
	EE4	I believe that using AI singing requires little effort.	
Perceived Agency (PA)	PA1	When using AI singing, I believe that I have control over the final generated singing outcome.	Taylor & Todd (1995); Belanche et al. (2022)
	PA2	When using AI singing, I believe that the AI-generated singing performance aligns with my expectations.	
	PA3	When using AI singing, I believe that my voice can influence the final output of the AI-generated singing.	
	PA4	When using AI singing, I believe that I clearly understand how my voice affects the AI-	

		generated singing results.	
Perceived Enjoyment (ENJ)	ENJ1	I feel that using AI singing is interesting.	Koufaris (2002); Moon & Kim (2001)
	ENJ2	I feel that using AI singing is enjoyable.	
	ENJ3	I feel that using AI singing is entertaining.	
	ENJ4	I feel that using AI singing stimulates my curiosity.	
Perceived Innovativeness (PI)	PI1	I believe that AI singing is distinctive.	Hwang et al. (2019)
	PI2	I believe that AI singing represents a new way of singing.	
	PI3	I believe that AI singing is creative.	
Ethical Acceptance (EA)	EA1	I believe that using AI singing for singing activities is ethically acceptable.	Peca et al. (2016)
	EA2	I believe that using AI singing in music-related applications is ethically appropriate.	
	EA3	I believe that recording and storing my voice data through AI singing is ethically acceptable.	
	EA4	I believe that AI singing using my voice for singing purposes is ethically acceptable.	
Affective Engagement (AE)	AE1	When using AI singing, I feel emotionally engaged.	O'Brien & Toms (2010)
	AE2	When using AI singing, I feel attracted to the experience.	
	AE3	When using AI singing, I feel involved in the entire process.	
Social Influence (SI)	SI1	People who are important to me think that I should use AI singing.	Venkatesh et al. (2003, 2012)
	SI2	People who influence my behavior think that I should use AI singing.	
	SI3	People whose opinions I value tend to prefer that I use AI singing.	

Continuance Intention (CI)	CI1	I intend to continue using AI singing rather than discontinue its use.	Bhattacherjee (2001, 2015); Venkatesh et al. (2012)
	CI2	Given the choice, I prefer to continue using AI singing rather than switch to alternative methods.	
	CI3	I plan to continue using AI singing in the future.	
